# Alternative polyadenylation regulates acetyl-CoA carboxylase function in peanut

**DOI:** 10.1186/s12864-023-09696-5

**Published:** 2023-10-24

**Authors:** Zhenying Peng, Shuang Yu, Jingjing Meng, Kai-Hua Jia, Jialei Zhang, Xinguo Li, Wenwei Gao, Shubo Wan

**Affiliations:** 1 Institute of Crop Germplasm Resources, Shandong Academy of Agricultural Science, Jinan, 250100 China; 2 College of Agricultural, Xinjiang Agricultural University, Urumqi, 830052 China; 3 Shandong Academy of Agricultural Science, Jinan, 250100 China

**Keywords:** Peanut, Alternative polyadenylation, Polyadenylation sites, 3′-untranslated region, Acetyl-CoA carboxylase, *AhACCA1* gene

## Abstract

**Background:**

Polyadenylation is a crucial process that terminates mRNA molecules at their 3′-ends. It has been observed that alternative polyadenylation (APA) can generate multiple transcripts from a single gene locus, each with different polyadenylation sites (PASs). This leads to the formation of several 3′ untranslated regions (UTRs) that vary in length and composition. APA has a significant impact on approximately 60–70% of eukaryotic genes and has far-reaching implications for cell proliferation, differentiation, and tumorigenesis.

**Results:**

In this study, we conducted long-read, single-molecule sequencing of mRNA from peanut seeds. Our findings revealed that over half of all peanut genes possess over two PASs, with older developing seeds containing more PASs. This suggesting that the PAS exhibits high tissue specificity and plays a crucial role in peanut seed maturation. For the peanut *acetyl-CoA carboxylase A1* (*AhACCA1*) gene, we discovered four 3′ UTRs referred to UTR1–4. RT-PCR analysis showed that UTR1-containing transcripts are predominantly expressed in roots, leaves, and early developing seeds. Transcripts containing UTR2/3 accumulated mainly in roots, flowers, and seeds, while those carrying UTR4 were constitutively expressed. In *Nicotiana benthamiana* leaves, we transiently expressed all four UTRs, revealing that each UTR impacted protein abundance but not subcellular location. For functional validation, we introduced each UTR into yeast cells and found UTR2 enhanced *AhACCA1* expression compared to a yeast transcription terminator, whereas UTR3 did not. Furthermore, we determined *ACC* gene structures in seven plant species and identified 51 PASs for 15 *ACC* genes across four plant species, confirming that APA of the *ACC* gene family is universal phenomenon in plants.

**Conclusion:**

Our data demonstrate that APA is widespread in peanut seeds and plays vital roles in peanut seed maturation. We have identified four 3′ UTRs for *AhACCA1* gene, each showing distinct tissue-specific expression patterns. Through subcellular location experiment and yeast transformation test, we have determined that UTR2 has a stronger impact on gene expression regulation compared to the other three UTRs.

**Supplementary Information:**

The online version contains supplementary material available at 10.1186/s12864-023-09696-5.

## Background

In eukaryotes, polyadenylation is a mechanism by which mRNA molecules are terminated at their 3′ ends. These appended polyadenylated (polyA) tails serve to safeguard mRNA against exonuclease degradation and are crucial for transcription termination, nuclear mRNA export, and translation [1, 2]. An alternative form of polyadenylation, known as alternative polyadenylation (APA), occurs when a locus generates multiple transcripts with distinct polyadenylation sites (PASs) during transcription. The 3′ untranslated region (UTR) plays a vital role in plant differentiation and development through alterations in protein length and diversity induced by APA of the encoding transcripts. Splicing and PAS are determinants of transcript length, with almost all eukaryotic mRNA undergoing transcription termination through processing of the 3′ end of the pre-mRNA during splicing and polyadenylation. Genomic investigations have revealed that over 50% of human genes are transcribed as a suite of pre-mRNAs with distinct PASs, resulting in diverse 3′ termini and a heterogeneous transcriptome and proteome [3].

APA can be categorized into two classes: untranslated region APA (UTR-APA) and coding region APA (CR-APA). UTR-APA shortens the 3′ UTR without changing the coding region, while CR-APA produces different protein isoforms by utilizing PASs within an intron [2]. In Arabidopsis, approximately 83% of APA events are UTR-APA, while the remaining 17% are CR-APA [4]. The composition and length of the 3’ end of different transcript variants can change without affecting the encoded protein sequence [1, 5]. Genes with high expression levels tend to have shorter 3’ UTRs, while genes with low expression levels tend to have longer 3’ UTRs, indicating that transcription may influence PAS selection. APA selection usually depends on the strength of sequence elements marking PAS, with long UTR sequences often containing classical sequence motifs such as AAUAAA [6]. Shorter 3′ UTRs can help mRNAs evade microRNA (miRNA) repression by removing the miRNA target site [7, 8]. Shorter 3’ UTRs produced by APA in mice improve transcript stability, potentially due to the presence of fewer miRNA target sites [7]. In addition, different 3’ UTRs may produce different secondary stem loop structures, which can also affect mRNA stability [9]. It has been shown that short 3’ UTRs can enhance translation efficiency of specific mRNAs [10]. Differences in the size of the 3’ UTR can also alter the location of neuronal mRNA [11].

APA occurs in approximately 70% of all human genes, regulating gene expression by modulating 3′ UTR length. This regulation influences the stability and translation efficiency of target mRNA, as well as the cellular localization of the resulting proteins. As a result, APA plays a fundamental role in various cellular processed such as proliferation, differentiation, and tumorigenesis. Recently, Li et al. constructed a multi-tissue atlas of human 3’ UTR-APA quantitative trait loci (3’aQTLs), containing approximately 400,000 common genetic variants associated with APA at target genes. These 3’aQTLs are distinct from other QTLs, such as expression QTLs, and significantly contribute to the molecular mechanisms underlying human complex traits and diseases [12]. APA is also involved in the regulation of important aspects of plant biology and physiology, including flowering, leaf development, pollen development, the circadian clock, and biotic and abiotic stresses. For example, over 60% of genes expressed in leaves undergo APA, with a lower extent of APA in younger leaves, but with more than 70% of PAS usages changing compared to cotyledons [13]. Biotic and abiotic stresses can also induce APA to produce different transcript subtypes. Under salt stress, the proportion of genes displaying APA significantly increased in Arabidopsis relative to saltwater cress (*Eutrema salsugineum*), indicating Arabidopsis’ sensitivity to salt stress compared to saltwater cress [14]. Hypoxia can result in transcripts with more PAS in the coding region [15]. Heat exposure induced more antisense PASs compared to cold exposure and control conditions, and a unique *cis*-element (AAAAAA) is predominately enriched downstream of PASs in *Populus trichocarpa* genes under the heat condition [16]. Alternative splicing (AS) and APA have been shown to regulate tissue senescence and dormancy of the fungus *Fusarium graminis* [17].

High-throughput sequencing technologies are making it easier to explore the full complement of transcripts in a cell. In particular, long-read sequencing provided by Oxford Nanopore Technology (ONT) significantly improves sequence read length compared to previous short-read sequencing platforms [18, 19]. Using high-throughput sequencing, more than 50% of all genes in eukaryotes have been shown to contain more than two PASs [1]. Many loci undergoing APA have been identified and studied to elucidate the biological effects of the resulting APA transcripts in specific tissues or developmental stages [20]. However, the potential role of APA in the regulation of fatty acid biosynthesis remains largely unknown.

In plants, Acetyl-CoA carboxylase (ACC) catalyzes the conversion of acetyl-CoA to malonyl-CoA. Overexpression of the subunit of heteromeric ACC from *Escherichia coli* in *Barssica napus* has been shown to increase the oil content of the transgenic seeds [21]. Similarly, overexpression of pea α-carboxyltransferase in Arabidopsis and camelina has been found to enhance fatty acid (FA) synthesis, thereby improving seed oil content [22]. Overexpression of four subunits encoding heteromeric *ACC* in upland cotton has also been reported to enhanced the oil accumulation in cotton seeds [23]. *ACC* genes have been identified in various plant species, including Arabidopsis, maize, wheat, rice, soybean, pea, alfalfa, rape and jatropha [23–27]. In our previous study, we identified 28 *AhACC* genes in the peanut genome, including 4 *ACCA*, up to 13 *ACCB*, 7 *ACCC*, 2 *ACCD*, and two homogenous *ACC* [28].

In this study, we performed ONT sequencing to analyze the peanut (*Arachis hypogaea* L.) transcriptome and identify genes with APA events. We cloned one of these genes, *Acetyl-COA carbosylase* (*ACC*), which encodes a key enzyme in plant FA biosynthesis, and discovered multiple 3′ UTRs for its transcripts. The expression pattern of these transcripts with different 3′ UTRs varied across different organs and stage of seed development. Through functional assays in budding yeast (*Saccharomyces cerevisiae*), we propose that APA plays a differential role in FA metabolism.

## Results

### Peanut seed developmental stages

**Fig. 1:**
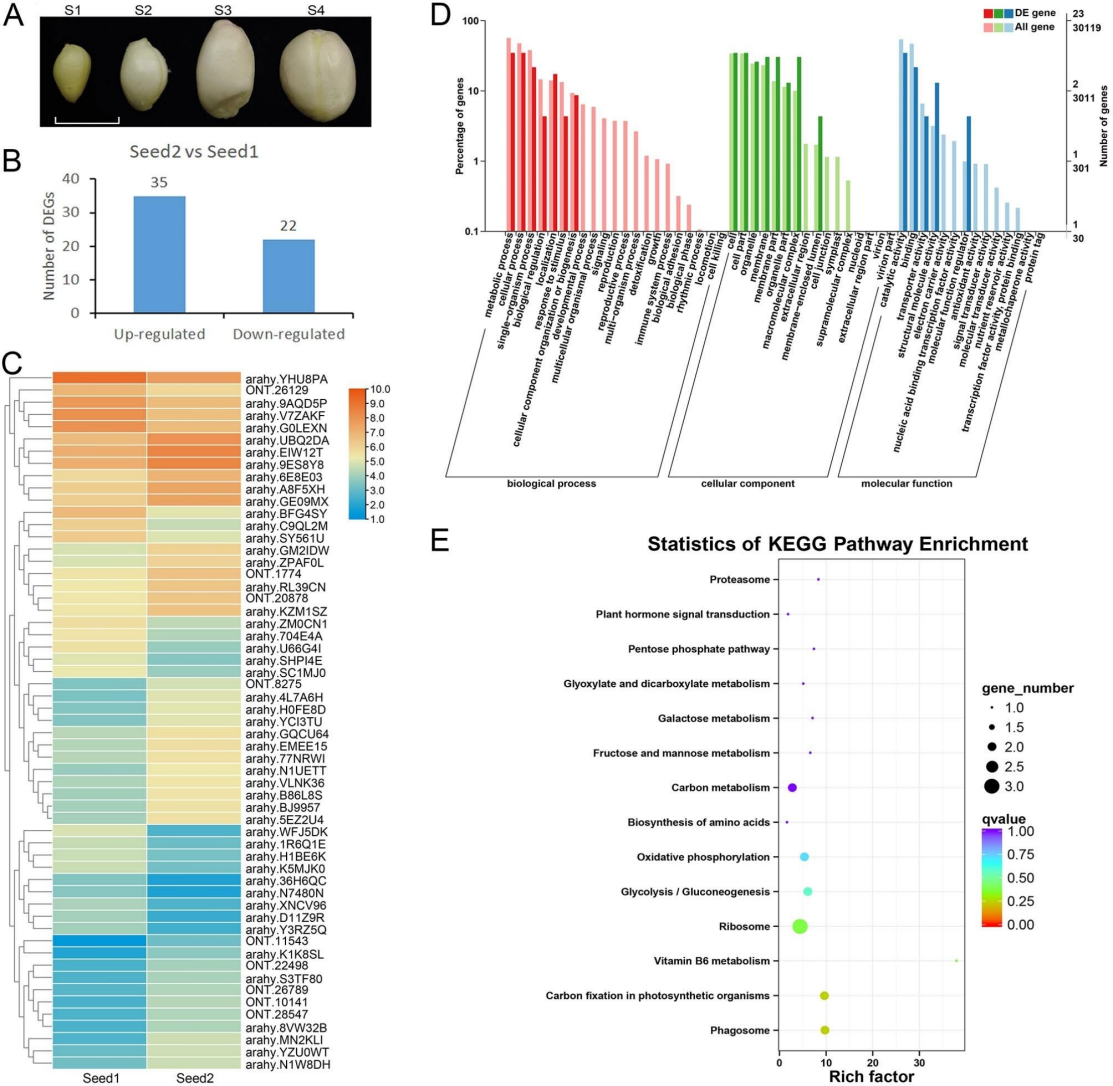
**Long-read sequencing data analysis of transcripts from developing peanuts**. (**A**) Representative photographs of the four developmental stages of peanut seeds, S1 to S4. Scale bar, 1 cm. (**B**) The number of upregulated and downregulated DEGs in Seed2 compared to Seed1. (**C**) Heatmap analysis of DEGs in Seed1 and Seed2. (**D**) GO enrichment analysis of the DEGs. (**E**) KEGG pathways enrichment of the DEGs. The KEGG pathway enrichment picture was drawn according to Kanehisa et al. (2022) [48]

Peanut seed developmental states were classified into four stages, designated S1 to S4 (Fig. 1A). S1 seeds displayed swelling, a smooth surface, a foxnut-like appearance with white spongy tissue, along with a small embryo. S2 seeds measured around 5–6 mm in length and exhibited a white episperm with a minute inner embryo. S3 seeds approximately 10 mm in length, with an immature embryo. S4 seeds reached a length of about 15–16 mm, with a fully mature embryo. The total RNA extracted from the seeds at S1 and S2 stages was labeled as Seed1, whereas the total RNA extracted from seeds at S3 and S4 stages was labeled as Seed2.

### ONT data collection and analysis

We sequence the Seed1 and Seed2 samples using ONT sequencing and generated sequencing libraries. After removing short and low-quality reads, we obtained 15.28 GB of clean, high-quality reads (Table S1). Using primers at both ends, we identified full-length coding sequences, resulting in 4,836,060 and 5,173,250 clean reads, respectively (Table S2).These full-length sequences were polished to obtain consensus isoform sequences. After aligning them to the peanut reference genome, we collapsed redundant sequences and obtained 48,435 non-redundant transcripts, representing 86.8–87.7% of full-length sequences (Table S2).

To analyze the transcriptional differences between Seed1 and Seed2, we compared their ONT transcriptomes and identified 57 significantly differentially expressed genes. Among these, 35 genes were upregulated in Seed2 compared to Seed1, while 22 genes were downregulated (Fig. 1B). Heatmap analysis confirmed these results (Fig. 1C). We performed GO term enrichment analysis and KEGG pathway enrichment analysis on the differentially expressed genes (DEGs). In the GO term analysis, the DEGs were assigned to three categories: biological process, cellular component and molecular function (Fig. 1D). In the “biological process” category, the genes were mainly involved in metabolic processes, cellular process and single-organism process, etc. In the “cellular component” category, the predominant groups were cell, cell part, organelle and membrane. In the “molecular function” category, the genes were distributed in catalytic activity, binding, transporter activity and structural molecule activity. The KEGG pathways analysis revealed pathways such as ribosome, carbon fixation in photosynthetic organisms, phagosome and glycolysis/gluconeogenesis (Fig. 1E).

**Fig. 2:**
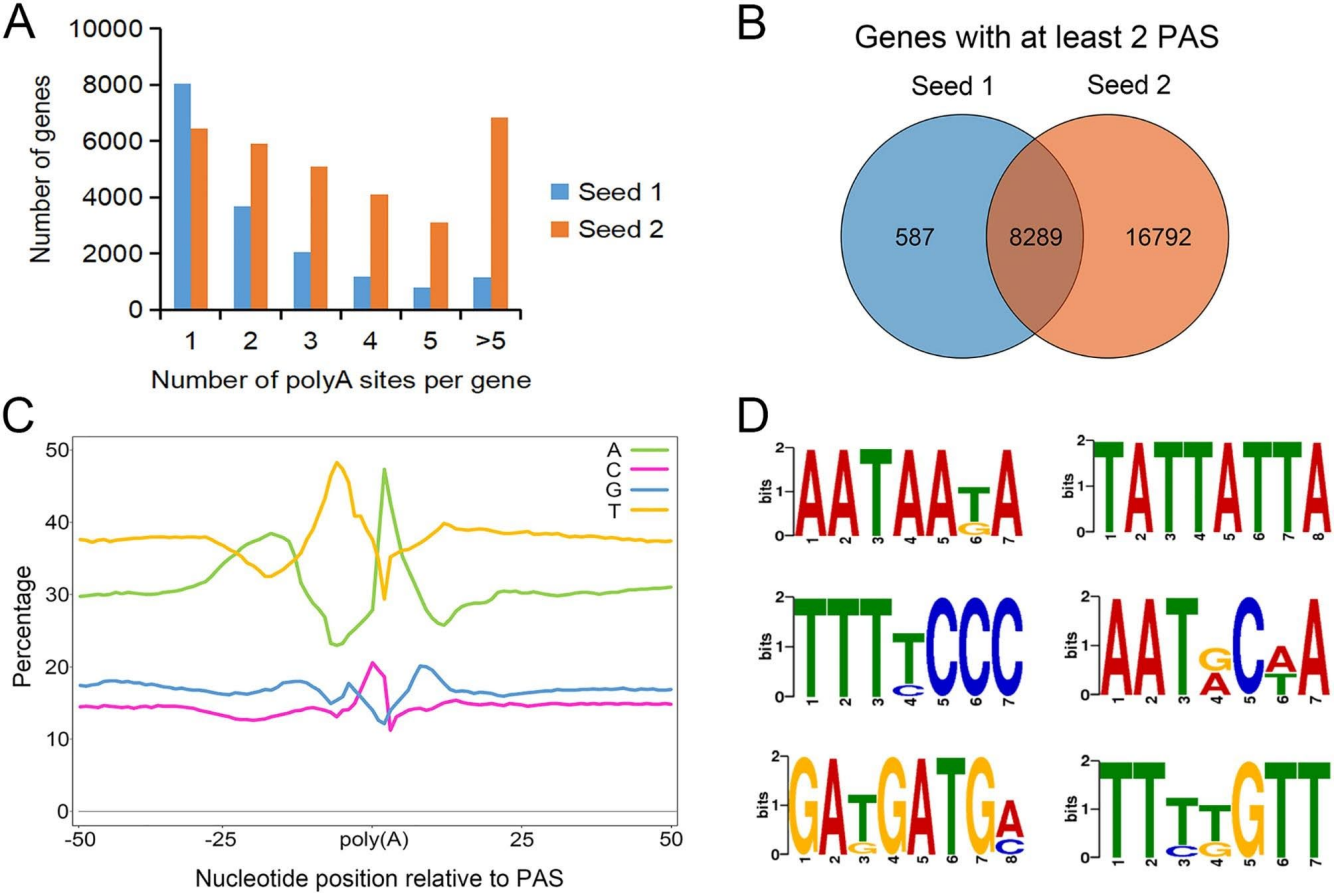
**APA analysis of the transcripts from Seed1 and Seed2**. (**A**) Distribution of the number of PASs per gene. Each PAS was required to be supported by at least two polyA reads, with at least 15 bp between two PAS clusters. (**B**) Venn diagram showing the extent of overlap between the number of genes with more than two PASs in Seed1 and Seed2. (**C**) Metaplot of the nucleotide composition over a 100-bp window centered around each PAS. (**D**) Sequence logos for some of the conserved motifs distributed near the PAS.

We also investigated PASs in the two seed samples separately. More than half of the expressed genes produced transcripts with at least two PASs: 8,876 in Seed1 and 25,081 in Seed2, which represents about 13.22% and 37.37% of all genes in the tetraploid peanut genome (Fig. 2A). Seed2 showed a higher number of PAS events compared to Seed1. We identified 587 genes with Seed1–specific PAS events and 16,792 genes with Seed2–specific PAS events, in addition to 8,289 genes shared between the two samples (Fig. 2B, Table S3-4). This finding indicates the developmental specificity of PASs. Analysis of nucleotide composition around the PAS revealed a sequence bias upstream and downstream of the PAS (Fig. 2C), suggesting that the authenticity of the identified polyA sites. By analyze 50-bp sequences upstream of the polyA sites, we identified 16 overrepresented motifs using TAPIS [29], such as AATAATA and TATTATTA (Fig. 2D).

### Amplification and structural gene analysis of UTRs of AhACCA1

**Fig. 3:**
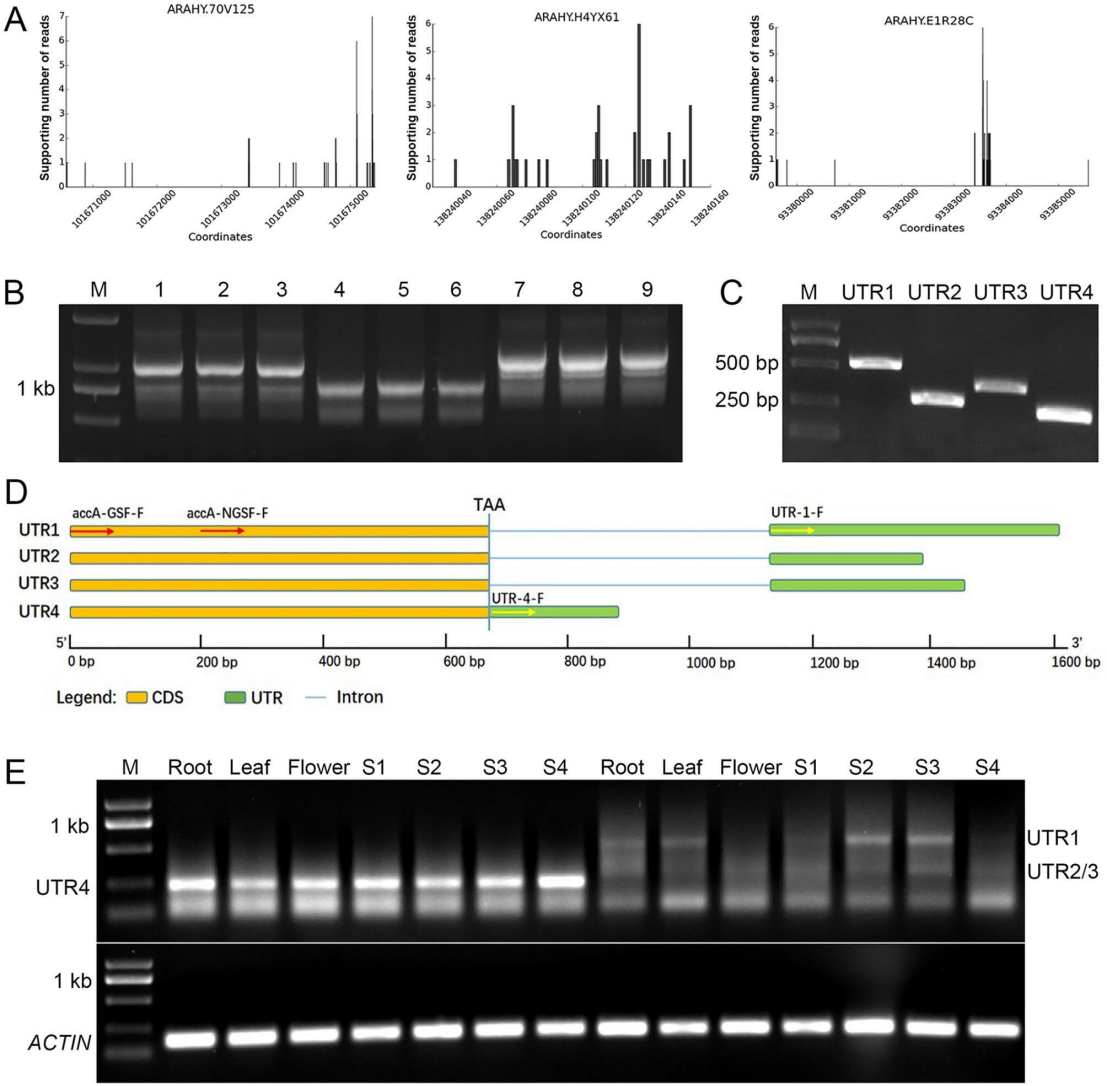
**Molecular identification of*AhACCA1*3**′ **UTRs**. (**A**) *AhACCA1*, *AhACCB1*, and *AhACCC1* produce transcripts with multiple polyadenylation sites. (**B**) 3′ RACE of UTRs for *AhACCA1*, *AhACCB1*, and *AhACCC1*. 1–3 lane: *AhACCA1*; 4–6 lane: *AhACCB1*; 7–9 lane: *AhACCC1*. M: marker. (**C**) PCR amplification of the four UTRs of *AhACCA1*. (**D**) Schematic diagrams of the four 3′ UTRs of *AhACCA1*. Red arrows indicate the forward primers of 3′ RACE, and yellow arrows indicate the forward primers for amplify the UTRs sequences. (**E**) RT-PCR analysis of UTR1–4. M, Trans 2 K plus DNA marker; S1–S4, the four different developmental stages of peanut seeds; *Actin11*, reference gene

We identified three *AhACC* genes with APA from ONT sequencing dataset (Fig. 3A). Arahy.70V125 corresponds to an *AhACCA* gene, arahy.H4YX61 to an *AhACCB* gene, and arahy.E1R28C to an *AhACCC gene*, which we designated as *AhACCA1*, *AhACCB1*, and *AhACCC1*, respectively. To characterize their 3′ UTRs, we performed a 3′ rapid amplification of cDNA ends (3′ RACE). Agarose gel electrophoresis of the PCR amplicons exhibited multiple bands for each reaction, suggesting the production of multiple transcript isoforms for each gene (Fig. 3B).

The 3’ RACE bands for *AhACCA1* were excised from the gel and after gel purification of the DNA, the 3′ RACE products were cloned and sequenced. Sequencing analysis aligned the results to the peanut reference genome, revealing the presence of four different 3’ UTRs for *AhACCA1*, which we named UTR1–4. UTR1 was 489 bp in length, UTR2 was 252 bp, UTR3 was 353 bp, and UTR4 was 211 bp (Fig. 3C, Fig. S1). Comparison of UTR1–4 with the peanut genome (Fig. 3D) showed that UTR1–3 contained a single intron that was identical in each UTR, with variations only in the sequence downstream of the intron. In contrast, UTR4 did not contain an intron and was the shortest among 3′ UTRs. Examination of the conserved PASs for each UTR revealed that UTR1 ended with AATAAA, UTR2 with ATTTG, UTR3 with ATTTTG, and UTR4 with TATAAA. To determine the expression pattern of each transcript isoform harboring UTR1–4, we performed RT-PCR analysis in three organs (roots, leaves, and flowers) and the four seed developmental stages (S1–S4) of peanut cultivar ‘Fenghua1’. Transcripts with UTR1 predominantly accumulated in roots and leaves during the S2 and S3 stages of seed development, while transcripts containing UTR2 or UTR3 were primarily present in roots and flowers across all four stages of seed development. Transcripts with UTR4 were highly expressed in all tissues (Fig. 3E). We propose that the observed differences in accumulation pattern may reflect distinct functions in peanut development.

### Functional analysis of UTR1–4 in *N. benthamiana* epidermal cells

**Fig. 4:**
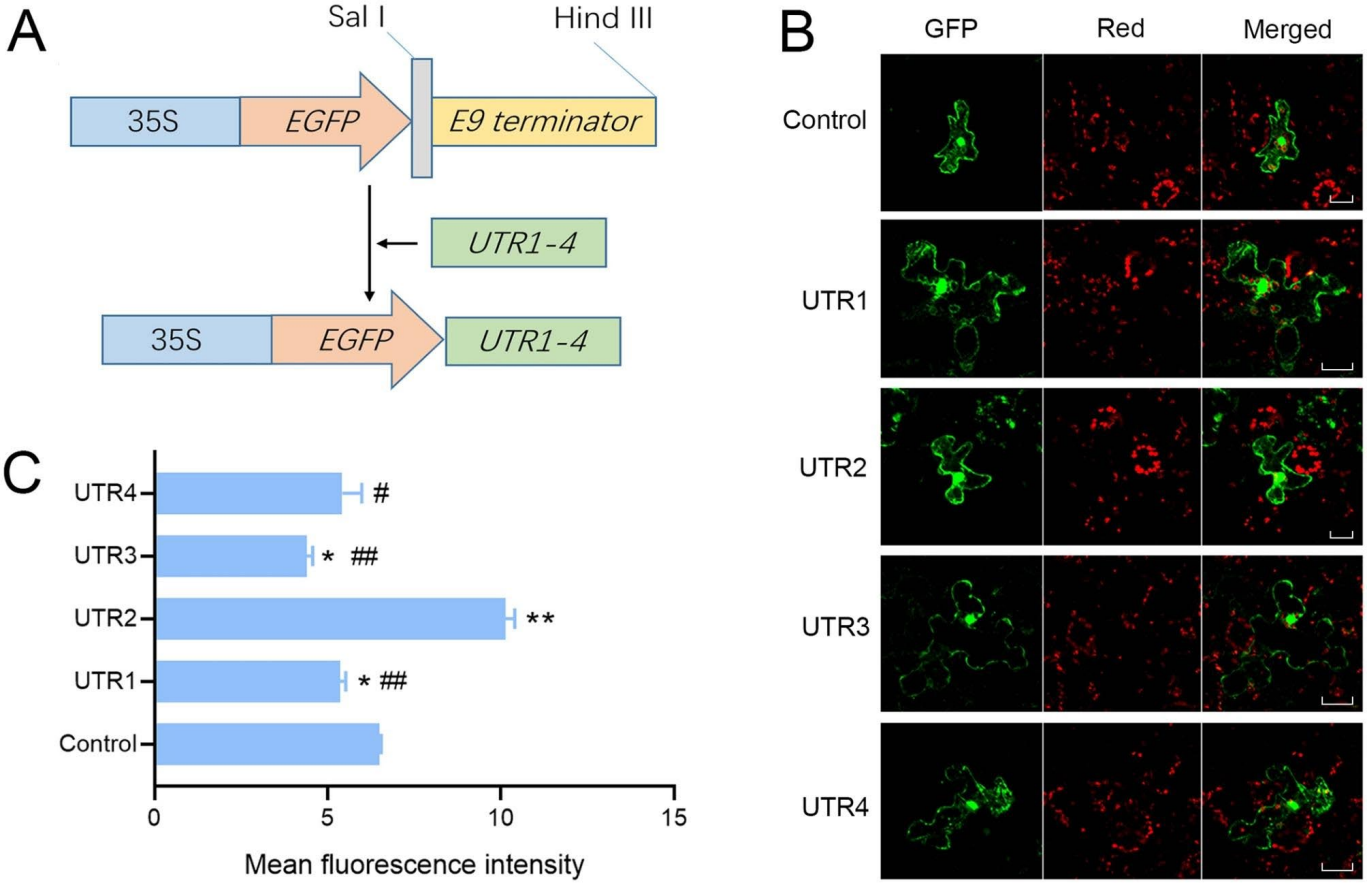
**Effect of the 3**′ **UTR from*AhACCA1*on protein localization and abundance in*Nicotiana benthamiana*epidermal cells**. (**A**) Schematic diagram of the constructs used to test the effect of each 3′ UTR from *AhACCA1* on GFP localization. (**B**) Representative confocal images of GFP fluorescence in *N. benthamiana* epidermal cells. Red, chlorophyll autofluorescence. Bar, 20 μm. (**C**) Quantification of fluorescence intensity from each transformation event shown in (B). Intensity was measured with ImageJ. UTR1–4 represent *EGFP:UTR1–4*. Control is the vector harboring the *E9* terminator. Significance analysis was performed by one-way ANOVA. Asterisks represent a significant difference compared to control; # represents a significant difference compared to *EGFP:UTR2*. *, *p* < 0.05; **, *p* < 0.01; #, *p* < 0.05; ##, *p* < 0.01

In order to assess the functional role of each 3′ UTR in regulating mRNA fate, we replaced the *E9* terminator in the pCAMBIA1300-35 S-EGFP vector with UTR1–4 from *AhACCA1* (Fig. 4A). Each resulting construct, designated as *EGFP:UTR1* to *EGFP:UTR4*, was transiently expressed *N. benthamiana* leaf epidermal cells. The control used was pCAMBIA1300-35 S-EGFP with the *E9* terminator. Fluorescence imaging revealed that all constructs displayed similar distribution patterns of green fluorescence, including the nucleus, cytoplasm, and plasma membrane, similar to the *35 S:EGFP* control (Fig. 4B). However, quantification of fluorescence intensity using ImageJ software revealed variations among constructs. *N. benthamiana* epidermal cells expressing *EGFP:UTR2* exhibited significantly higher fluorescence intensity compared to the control (*p* < 0.01), while cells expressing *EGFP:UTR1* and *EGFP:UTR3* displayed lower fluorescence intensity than the control (*p* < 0.05). The fluorescence intensity of *EGFP:UTR4* was comparable to that of the control (Fig. 4C). Furthermore, when comparing *EGFP:UTR1* and *EGFP:UTR3* to *EGFP:UTR2*, significantly lower fluorescence intensity was observed (*p* < 0.01). Additionally, *EGFP:UTR4* exhibited significantly lower fluorescence intensity compared to *EGFP:UTR2* (*p* < 0.05) (Fig. 4C). These findings suggest that the four UTRs of *AhACCA1* likely impact protein synthesis, but have minor influence on subcellular protein location.

### Fatty acid composition analysis of yeast strains harboring each 3′ UTR

**Fig. 5:**
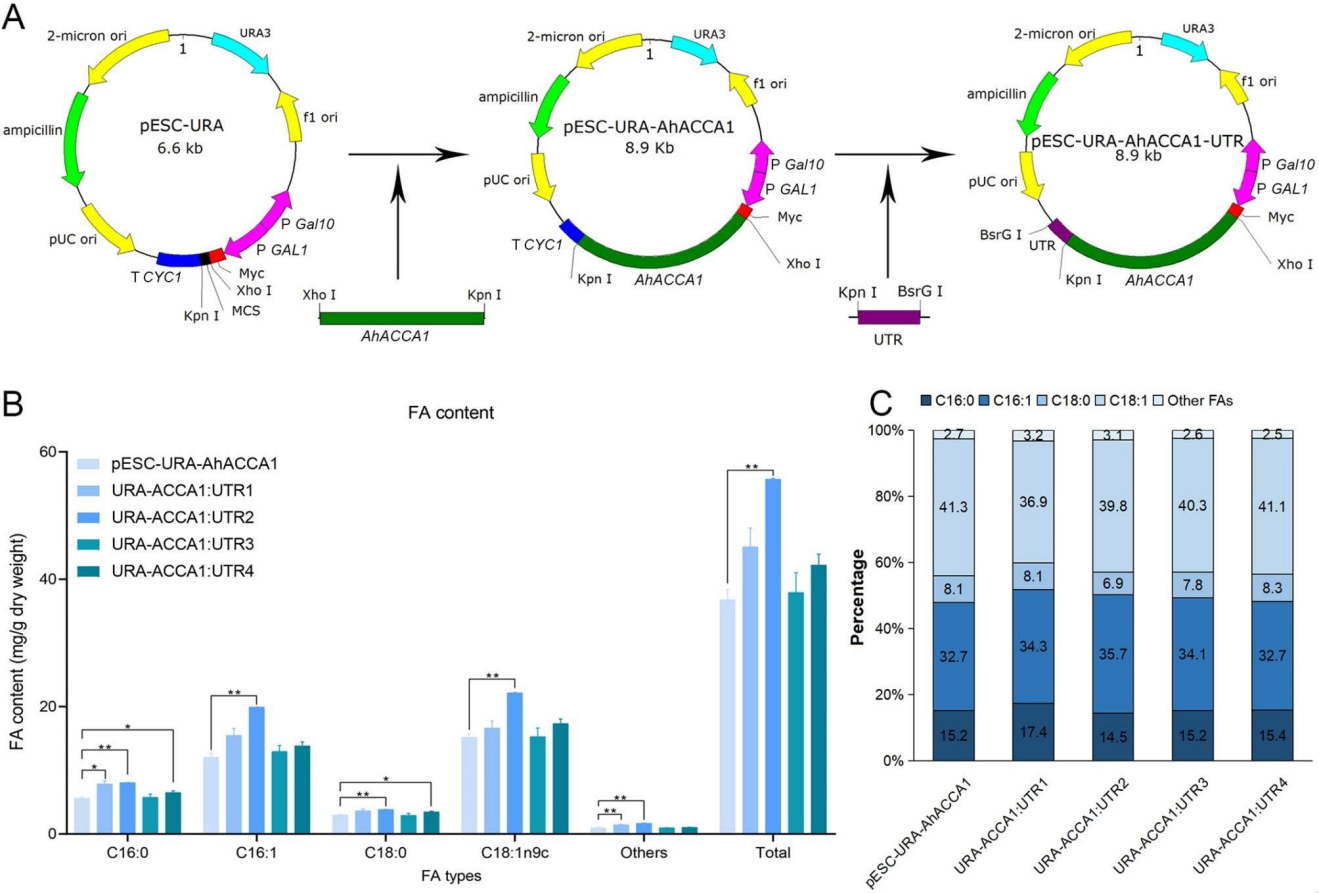
**FA content and composition of transgenic yeast strains**. (**A**) Schematic diagram of the constructs used to test the effect of each 3′ UTR from *AhACCA1* on fatty acid content in yeast. (**B**) Mean FA content in the indicated transformed yeast colonies. C, Relative FA composition. Significance analysis was performed by two-way ANOVA. *, *p* < 0.05; **, *p* < 0.01

*AhACCA1*, a 2,262 bp open reading frame (ORF), was cloned into the yeast vector pESC-URA, resulting in the plasmid pESC-URA-AhACCA1. To investigate the effect of different 3’ UTRs on the expression of AhACCA1, we individually replaced the *CYC1* terminator with UTR1–4, generating four plasmids: URA-ACCA1:UTR1, URA-ACCA1:UTR2, URA-ACCA1:UTR3, and URA-ACCA1:UTR4 (Fig. 5A). We separately transformed each plasmid into yeast strain W303 and screened for positive transformants. The control plasmid pESC-URA-AhACCA1 was used as the reference.

The OD_600_ value of the yeast cultures was monitored every 12 h, and a growth curve was generated to assess the impact of each UTRs on the growth rate of the transformed yeast cells (Fig. S2). The growth curves of all transformants were similar, with a lag phase from 0 to 36 h, a logarithmic growth phase from 36 to 60 h, and a stationary phase at 72 h. The most significant difference in growth rate among the transformants was observed during the 48 to 60 h stage, where UTR2 transformant exhibited the highest growth rate, while UTR3 transformants exhibited the lowest. During the stationary phase, differences between the transformants were not apparent.

After 84 h of culture, the yeast cell were collected for fatty acid content analysis using GC-MS [30]. We analyzed the content and composition of four major fatty acid in yeast: palmitic acid (C16:0), palmitoleic acid (C16:1), stearic acid (C18:0), and oleic acid (C18:1).

The *URA-ACCA1:UTR2* transformants displayed 28.8–51.4% higher level of C16:0, C16:1, C18:0, and C18:1, as well as total fatty acid content, compared to yeast carrying the control plasmid pESC-URA-AhACCA1 (Fig. 5B). This suggests that UTR2 has some influence on the abundance of AhACCA1 compared to the *CYC1* terminator. In contrast, the fatty acid content of yeast strains harboring the *URA-ACCA1:UTR3* plasmid did not differ significantly from that of the control, indicating that UTR3 has little effect on AhACCA1 abundance in yeast. Additionally, UTR2 influenced the fatty acids composition of the transformed yeast strain, decreasing the ratio of C16:0, C18:0, and C18:1 while increasing the ratio of C16:1 (Fig. 5C). This result indicates that UTR2 impacts the abundance of the translated protein derived from the transcript.

### *Cis*-acting elements within UTR1–4

**Fig. 6:**
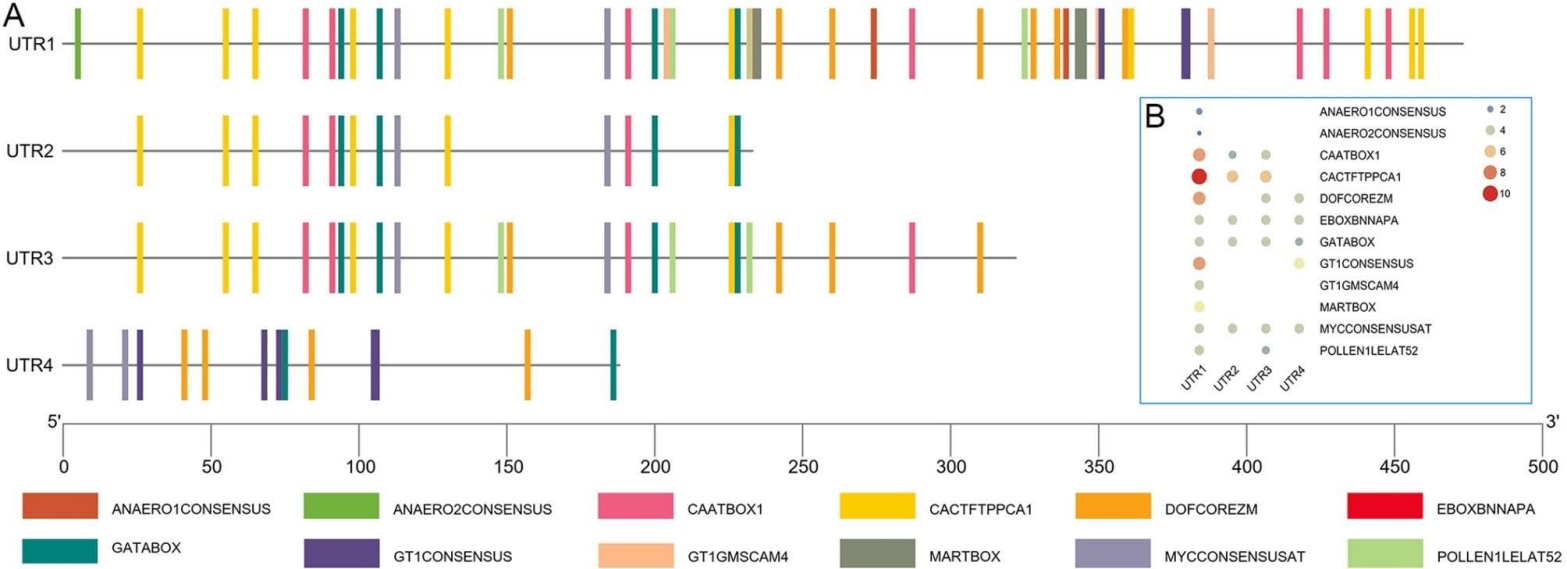
***Cis*-elements in UTR1–4 of*AhACCA1***. (**A**) Distribution of *cis*-elements in UTR1–4. (**B**) Number of motifs present in each 3′ UTR

The 3′ UTRs of transcripts play a crucial role in post-transcriptional regulation. Specifically, *cis*-elements within 3′ UTRs can interact with RNA-binding proteins in a sequence-specific or structure-dependent manners, allowing for the regulation of mRNA stability, translation, and localization. The four possible 3′ UTRs of *AhACCA1* vary in their length and sequence, indicating that their *cis*-elements are likely to be partially distinct. Therefore, we conducted an analyzed of the motifs present in four UTRs using TBtools software [31]. UTR1, which is the longest 3′ UTR, contains the highest number of motifs, followed by UTR3 and UTR2 (Fig. 6A). Furthermore, the types of motifs present also differ among the 3′ UTRs. UTR1 harbors the highest number of motif types (about 12), while UTR2 and UTR4 have a lower number of motif types (only 5, Fig. 6B). Some motifs, such as EBOXBNNAPA, GATABOX, and MYCCONSENSUSAT, were present in all four 3′ UTRs. However, motifs such as ANAERO1CONSENSUS, ANAERO2CONSENSUS, GT1GMSCAM4, and MARTBOX are exclusive to UTR1. The presence of fewer motifs in UTR2 and UTR4 suggests a lower degree regulation by RNA-binding proteins and/or under specific expression conditions. We propose that the enhanced translation observed for UTR2, as indicated by the increased EGFP fluorescence intensity in *N. benthamiana* cells and higher FA biosynthesis in yeast, can be attributed to its shorter length and fewer motifs.

## Discussion

Transcription proceeds through the stages of initiation, extension, and termination. While a strong promoter is essential for high-level gene expression, transcription terminators are equally important. 3’ UTRs are unique sequence structures that play an important role in the regulation of transcription and mRNA stability in eukaryotes. In fact, more than half of all human genes are reported to use alternative splicing and alternative polyadenylation to generate mRNA isoforms that differ only in their 3′ UTRs [32]. Deep 3′ sequencing data from 23 developmental stages, tissues, and cell lines of the fruit fly *Drosophila melanogaster* revealed approximately 62,000 PAS, two-thirds of which undergo APA [33]. In *Fusarium graminearum*, 11,133 genes were identified to have alternatively polyadenylated transcripts [17]. In Arabidopsis, over 60% of genes expressed in leaves undergo APA, with evidence of developmental stage–specific regulation [13]. In our study, we identified 8,876 genes in Seed1 and 25,081 gene in Seed2 that possess at least two PASs (Fig. 2A, Table S3-4), accounting for about 79.02% of all expressed genes (32,483 genes) in the Seed1 and Seed2 samples. These results indicate that genes with multiple PAS events are involved in specific biological responses, including metabolism and cellular programs, shedding new insight on the regulation of genes expression through APA.

All APA sites are located in the 3′ UTRs, resulting in mRNA isoforms with differing lengths and sequences in their 3′ UTRs. Studies of 3′ end sequencing data from diverse tissues have shown that the positioning of functional PASs is consistent across cell types, but the expression levels of alternative 3′ UTR isoforms are highly tissue and cell type specific [32]. In Chinese tulip tree (*Liriodendron chinense*), 6,721 genes with APA sites were identified, 187 of which showed tissue-specific expression [34]. Investigating the function of isoforms with distinct 3′ UTRs is challenging because the derived amino acid sequences of the encoded proteins are identical. However, the 3′ UTR sequence may contain additional genetic information that distinguishes the functions of the encoded proteins [35]. In peanut seeds, genes with more than two PASs exhibited significant tissue-specific expression pattern. We observed more PASs in the Seed2 sample (mature stage) compared to the Seed1 sample (young stage, Fig. 2A, B), suggesting the important role of PASs in peanut seed development and maturation. Furthermore, the four types of *AhACCA1* transcripts with different 3′ UTRs showed tissue-specific expression patterns (Fig. 3E), suggesting that the proteins encoded by each transcript isoform may exert distinct functions in peanut leaves, roots, and during seed development.

Each 3’ UTR, located at the end of the transcriptional unit, plays a crucial role in regulating the expression and contains specific functional elements that impact the efficiency of gene expression. The *c-fos* gene was the first example of a 3′ UTR with specific functional elements. It was found that absence of the 3′ UTR allowed *c-fos* to transform fibroblasts, indicating the destabilization of c-fos transcripts through AU-rich elements in the 3’ UTR [36, 37]. Interestingly, AU-rich elements are sometimes more conserved than the coding regions of genes and are believed to recognition signal for mRNA degradation of certain genes [38, 39]. In addition, AU-rich elements have been shown to enhance protein production by interacting with specific *trans*-acting factors that bind to the *cis*-elements within 3′ UTR [40, 41]. In our study, we identified multiple *cis*-elements in the 3′ UTRs of *AhACCA1*. Some of these elements (such as EBOXBNNAPA, GATABOX, and MYCCONSENSUSAT) were shared by all 3′ UTRs, while others (such as ANAERO1CONSENSUS, ANAERO2CONSENSUS, GT1GMSCAM4, and MARTBOX) were specific to certain 3′ UTRs (Fig. 6). These variations in cis-elements may influence translation efficiency or mRNA stability. Results from transient expression of *EGFP* constructs in *N. benthamiana* leaf epidermal cells and stable expression in yeast cells demonstrated that the shorter UTR2, which contains fewer motifs compared to the other three 3′ UTRs of *AhACCA1*, is associated with high expression level, highlighting the significant impact of UTR length and motifs.

To maximize expression, careful selection of transcription terminators (3′ UTRs) has proven to be essential in plant expression vectors. Currently, widely used 3′ UTRs include those from the *Nopaline synthase* (*NOS*) or *Octopine synthase* (*OCS*) genes from Agrobacterium, as well as the 35 S terminator from cauliflower mosaic virus [9]. However, various studies have shown that plant 3′ UTRs have a greater potential to enhance expression compared to *NOS*, *OCS*, or 35 S 3′ UTRs. For example, in alfalfa (*Medicago sativa*), tobacco (*N. tabacum*), and *N. benthamiana*, the use of the 3′ UTR from *Ribulose-1,5-bisphosphate carboxylase* (*rbcS*) resulted in higher expression levels from reporter genes compared to the *NOS* terminator. Similarly, in rice seeds, the expression of mite allergen (mDer f 2) was four times higher when using the 3′ UTR of *glutelin B-1* (*GluB-1*, rice) compared to the *NOS* terminator [42]. Other rice 3′ UTRs, such as *GluB-5*, *GluA-2*, and *GluC*, also showed higher expression levels compared to the *NOS* terminator [43]. Another popular 3′ UTR is from the *HEAT SHOCK PROTEIN* (*HSP*) gene, which confers high expression levels to target genes in both monocot and dicot species compared to *NOS*, *OCS*, or 35 S terminator. Furthermore, certain plant 3′ UTRs, including *ALCOHOL DEHYDROGENASE* (*ADH*), *histone H4* (*H4*), and *UBIQUITIN 5* (*UBQ5*), have demonstrated even greater potential for driving high expression levels than NOS, OCS, or 35 S terminator [44]. In our study, we identified four 3′ UTRs for the *AhACCA1* locus. Both transient and stable expression assays revealed that these four 3′ UTRs exhibited different expression levels, with UTR2 demonstrating the highest expression compared to the other three 3′ UTRs from *AhACCA1*, as well as compared to pea *E9* (derived from *RbcS*) and yeast *CYC1* terminator. Conversely, UTR1 and UTR3 showed lower expression level than the control terminator (Fig.s 4 and 5). Therefore, appropriate select of a suitable transcription terminator is necessary to ensure optimal expression of target genes.

Transcription terminator mechanisms in plant have been extensively studied in plants, but there has been limited research on those derived from FA biosynthesis genes. As peanut is an important oil crop, it is crucial to investigate the regulatory mechanism of its oil biosynthetic genes. The primary substrate for FA biosynthesis is acetyl-CoA, which is catalyzed by ACC. Therefore, the enzyme activity of ACC directly influences FA biosynthesis and oil content. Our finding indicate that *AhACCA1*, a gene associated with FA biosynthesis in peanuts, possesses four 3′ UTRs, each appearing to have an impact on mRNA stability or translation efficiency. These discoveries provide novel insights into the regulation of *AhACCA1* expression. To extend our understanding, we examined whether *ACC* genes in other plant species undergo APA. Information for 15 *ACC* genes with APA was extracted from four plant species: five in Arabidopsis, one in rice, two in *T. pratense*, and seven in *M. truncatula* (Table S5). We observed that APA of *ACC* genes is widespread across various plant species. Therefore, unravelling the regulatory mechanism of 3’ UTRs from *AhACCA1* will not only help elucidate how *ACC* gene expression is regulated in peanut but also in other plant species.

## Conclusions

In this study, total RNA was extracted from developing peanut seeds and subjected to long-read-based ONT sequencing to identify the genes with APS in peanut seeds. Four 3′ UTRs from *AhACCA1* were tested using RT-PCR, revealing differential expression levels in different organs for each corresponding transcript isoforms. The transient expression of each 3′ UTR cloned downstream of *EGFP* in *N. benthamiana* leaf epidermal cells and the stable expression in yeast cells downstream of the *AhACCA1* coding sequence demonstrated that each 3′ UTR had a distinct influence on the targeted genes, with UTR2 displaying a strong ability to support high expression levels. The presence of APS was found to be widespread among *ACC* genes, rather than being specific to peanut, indicating that its broad involvement in plant fatty acid biosynthesis.

## Materials and methods

### Peanut seed preparation and RNA extraction

The developmental process of peanut seeds was divided into four stages: S1-S4. The total RNA extracted from seeds at each stages and mixed equal amounts of total RNAs from seeds at S1 and S2 stages, yielding the Seed1 sample, and equal amounts of total RNAs from seeds at the S3 and S4 stages to obtain the Seed2 sample. RNA was extracted using the TRIzol reagent (Invitrogen, Carlsbad, CA, USA) then treated with RNase-free DNase I (New England Biolabs, USA) for 30 min at 37 °C to degrade any contaminating DNA present. The concentration and purity of the resulting RNA preparations were assessed using a NanoDrop 2000 spectrophotometer (Thermo Fisher Scientific, Wilmington, DE, USA) and its integrity was checked using an RNA Nano 6000 Assay Kit (Agilent Technologies, CA, USA).

### ONT sequencing and data analysis

cDNA libraries were constructed using a cDNA-PCR Sequencing Kit (SQK-PCS109) following the protocol provided by Oxford Nanopore Technologies (ONT). Briefly, the template-switching activity of reverse transcriptase was used to enrich for full-length mRNAs. Defined PCR adapters were added directly to both ends of the first-strand cDNA, followed by amplification by PCR for 14 circles with LongAmp Tag (NEB). The PCR products were then subjected to ONT adapter ligation using T4 DNA ligase (NEB). Agencourt XP beads were used for DNA purification according to the ONT protocol. The final cDNA libraries were loaded onto a FLO-MIN109 flowcell for sequencing on a PromethION platform from Biomarker Technology Company (Beijing, China). Raw reads were filtered for a minimum average read quality score of 7 and a minimum read length of 500 bp. Ribosomal RNA was discarded after mapping to the rRNA database (http://www.peanutbase.org). Next, full-length, non-chimeric (FLNC) transcripts were determined by searching for primers at both ends of the reads. Clusters of FLNC transcripts were obtained after mapping to the peanut reference genome (http://www.peanutbase.org) with mimimap2 [45], and consensus isoforms were obtained after polishing within each cluster by pinfish. Consensus sequences were mapped to the reference genome using minimap2. Mapped reads were collapsed by the cDNA_Cupcake package with min-coverage = 85% and min-identity = 90%.

Transcripts were validated against known annotation files for peanut with gffcompare [46]. APA analysis was conducted with TAPIS [29]. Coding sequences were predicted by TransDecoder [47]. Functional gene annotation was performed based on the following databases: NR (NCBI non-redundant protein sequences), Pfam (Protein family), KOG/COG/eggNOG (Clusters of Orthologous Groups of proteins), Swiss-Prot (A manually annotated and reviewed protein sequence database), KEGG (Kyoto Encyclopedia of Genes and Genomes) [48], and GO (Gene Ontology).

Full-length reads were mapped to the reference transcriptome (http://www.peanutbase.org). Reads with match quality above 5 were used for quantification. Expression levels were estimated based on number of reads per gene/transcript per 10,000 reads mapped. Prior to differential gene expression analysis, for each sequenced library, the read counts were adjusted with the R package edgeR through one scaling normalized factor [49]. Differential expression analysis between two samples was performed using edgeR (3.8.6). A false discovery rate (FDR) < 0.01 and a fold-change ≥ 2 were set as the threshold for significant differential expression.

### RACE amplification

Total RNA was extracted from the seeds of peanut cultivar ‘Fenghua 1’ with RiboPure RNA Purification Kits (ThermoFisher) and reverse transcribed into first-strand cDNA using a HiScript-TS 5′/3′RACE Kit (Vazyme Biotechnology Co., LTD, Nanjing, China) according to the manufacturer’s manual.

### Cloning of the AhACCA1 coding sequence

The coding sequence of *AhACCA1* was amplified by PCR using primers accA1-F and accA1-R (Table S6). Each 25-µL reaction contained 1 µL cDNA (100 ng/µL) from Seed1, 1 µL of each primer (10 µM), 12.5 µL 2× Phanta® Max Master Mix (Dye Plus), and 9.5 µL ddH_2_O. The amplification program comprised an initial denaturation (95 °C for 3 min), followed by 30 cycles of 95 °C for 15 s, 60 °C for 15 s, and 72 °C for 90 s, with a final elongation of 72 °C for 5 min. The amplicons were resolved by electrophoresis on a 1% (w/v) agarose gel, gel purified with a PureLink™ PCR Purification kit (ThermoFisher), and sequenced by Sangon Biotech (Shanghai, China).

### Cloning of the 3′ UTRs of AhACCA1

Nested PCR was conducted to amplify the 3′ UTRs of *AhACCA1* using primers UTR-1-F, UTR-2-F, and UTR-R (Table S6). The fragments were subcloned into the pCE2 TA/Blunt-Zero vector (Vazyme, Nanjing,China) for sequencing. The 3’ UTRs were compared with the corresponding genomic sequence to determine gene structure using the online software GSDS 2.0 [50].

### RT-PCR tests of the 3′ UTRs in different tissues

Total RNAs from young roots, leaves, flowers, and the seeds of S1–S4 stages of peanut cultivar ‘Fenghua 1’ were isolated using the TRIzol reagent (Invitrogen, Carlsbad, CA, USA) and reverse transcribed into first-strand cDNAs using the FastPure Plant Total RNA Isolation Kit (Vazyme Biotechnology Co., LTD, Nanjing, China) according to the manufacturer’s manual. The cDNAs were diluted to the same concentration, and the peanut *Actin* gene (Actin-F and Actin-R, Table S6) was used as the reference. Primers UTR-1-F, UTR-4-F, and UTR-R were used to amplify the fragments of 3′ UTRs from different organs. Each 20-µL reaction consisted of 1 µL cDNA (100 ng/µL), 1 µL of each primer (10 µM), 10 µL 2×Taq Master Mix, and 7 µL RNase-free H_2_O. The amplification program comprised an initial denaturation (95 °C for 1 min), followed by 30 cycles of 95 °C for 15 s, 60 °C for 15 s, and 72 °C for 40 s, with a final elongation of 72 °C for 5 min. The amplicons were resolved by electrophoresis on a 1.2% (w/v) agarose gel.

### Subcellular localization of proteins encoded by AhACCA1 transcripts

Full-length sequences of the four 3′ UTRs of *AhACCA1* were amplified using primers (GFP-1-F-Sal I, GFP-1-R-Hind III, GFP-2-R-Hind III, GFP-3-R-Hind III, GFP-4-F-Sal I, and GFP-4-R-Hind III, Table S6) containing the sites for the restriction enzymes *Sal* I and *Hind* III and cloned into the pCAMBIA1300-35S-EGFP vector. The E9 terminator of EGFP was replaced to verify the functionality of the 3’ UTRs. The resulting clones were named *EGFP-UTR1*, *EGFP-UTR2*, *EGFP-UTR3*, and *EGFP-UTR4* and were individually transformed into Agrobacterium (*Agrobacterium tumefaciens*) strain GV3101 according to Gui et al. [51]. The appropriate cell density of each bacterial culture was infiltrated into *Nicotiana benthamiana* epidermal cells. The plants were placed in the dark for 1 day and then in dysphotic conditions (illumination intensity: ~50 lx) for 1–2 days before peeling off the leaf abaxial epidermis for observation using a confocal microscope (Leica TCS-SP8 SR, German). An Agrobacterium suspension harboring the pCAMBIA1302-35 S-EGFP empty vector was used as the control.

### Quantification of EGFP-fluorescence intensity

The EGFP-fluorescence intensity was quantified with ImageJ software with default parameters [52].

### Yeast genetic transformation and fatty acid tests

The full-length coding sequence of *AhACCA1* was amplified using primers (URA-accA1-Xho-F I and URA-accA1-KpnI-R, Table S6) and cloned into the multiple cloning site of the pESC-URA vector by double digestion with *Xho* I and *Kpn* I. The recombinant vector was named pESC-URA-AhACCA1.

The four 3′ UTRs were amplified using specific primers (URA-1-F-Kpn I, URA-1-R-BsrG I, URA-2-R-BsrG I, URA-3-R-BsrG I, URA-4-F-Kpn I, and URA-4-R-BsrG I, Table S6) and inserted into the above pESC-URA-*AhACCA1* vector by double enzyme digestion with *Kpn* I and *BsrG* I to replace the original *CYC1* terminator. The resulting clones were named URA-ACCA1:UTR1, URA-ACCA1:UTR2, URA-ACCA1:UTR3, and URA-ACCA1:UTR4. The recombinant vector pESC-URA-AhACCA1 with the *CYC1* terminator was used as control. All clones were individually transformed into yeast strain W303 and selected transformants on synthetic defined (SD) medium lacking Ura (SD-URA) for about 3–5 days. OD_600_ value of the yeast broth was detected every 12 h and yeast growth curve was plotted using Excel. Positive transformants cultured for 84 h were collected for fatty acid tests by gas chromatography–mass spectrometry (GC-MS) [30].

### Fatty acid analysis

Yeast cells were cryodesiccated, accurately quantified and ground into a powder in a test tube. Samples were soaked in 2 mL of 2% sulfuric acid in dry methanol for 16 h at room temperature, followed by 80 min of heating at 90 °C to convert the FAs into FA methyl esters (FAMEs). Supelco™ 37 Component FAME Mix (Sigma-Aldrich Company, Missouri, USA) was added to the samples as an internal standard. After addition of 2 mL of distilled water and 3 mL of hexane, the FAMEs were extracted for analysis by GC-MS.

The FAME composition of the samples was analyzed using Agilent Technologies 6890 N gas chromatograph (Agilent Company, California, USA), with a programmed temperature gas chromatography method for lipid measurement. An initial column temperature of 140 °C was maintained for 5 min then increased to a final temperature of 240 °C at a rate of 4 °C/min, held for 10 min. Injection and detector temperatures were 240 °C and 26 °C, respectively. Two microliters of each sample were injected into the column. FAMEs were identified by comparison of their retention times with those of the standards. The results were analyzed using the Chrom Perfect® LSi system chromatography software (Chrom Perfect® LSi system, UK) with the FAME mix peak area as the reference.

FA content was computed as absolute content (mg/g) using the GC area counts for the different FAMEs. The quantities of the FAMEs in each sample were used to calculate oil content using the equation:

$$Wi = \frac{{Ai*Ms}}{{As*M}}$$

Where M_s_ is the weight of the internal standard added to a sample, A_i_ is the area counts of the individual FAME, A_s_ is the area count of the corresponding FAME in the internal standard, and M is the weight of the sample used. Two repeats were conducted for each sample.

### Analysis of cis-acting elements in UTR1–4

The analysis of *cis*-acting elements in UTR1–4 was performed using the online server PlantCARE ( http://bioinformatics.psb.ugent.be/webtools/plantcare/html/) [53]. The distribution of motifs in each UTR was drawn using TBtools [54].

### Comparison of PASs of other plant species

The keyword “Acetyl-CoA Carboxylase” was entered on the website PlantAPAdb (http://www.bmibig.cn/plantAPAdb/) and searched in the databases of seven plant species: rice (*Oryza sativa* L. [*japonica* and *indica*]), Arabidopsis (*Arabidopsis thaliana*), *Chlamydomonas reinhardtii*, barrel clover (*Medicago truncatula*), red clover (*Trifolium pratense*), black cottonwood (*Populus trichocarpa*), and Moso bamboo (*Phyllostachys edulis*) [55].

### Electronic supplementary material

Below is the link to the electronic supplementary material.

Supplementary Material 1

Supplementary Material 2

Supplementary Material 3

Supplementary Material 4

Supplementary Material 5

Supplementary Material 6

## Data Availability

The datasets used and/or analysed during the current study available from the corresponding author on reasonable request. The collection and handling of plant were in accordance with all the relevant guidelines.
