# Supplementary figures and images for: Alternative polyadenylation regulates acetyl-CoA carboxylase function in peanut
Source: BMC Genomics. 2023 Oct 24;24:637. doi: 10.1186/s12864-023-09696-5 (PMC10594767; doi:10.1186/s12864-023-09696-5)

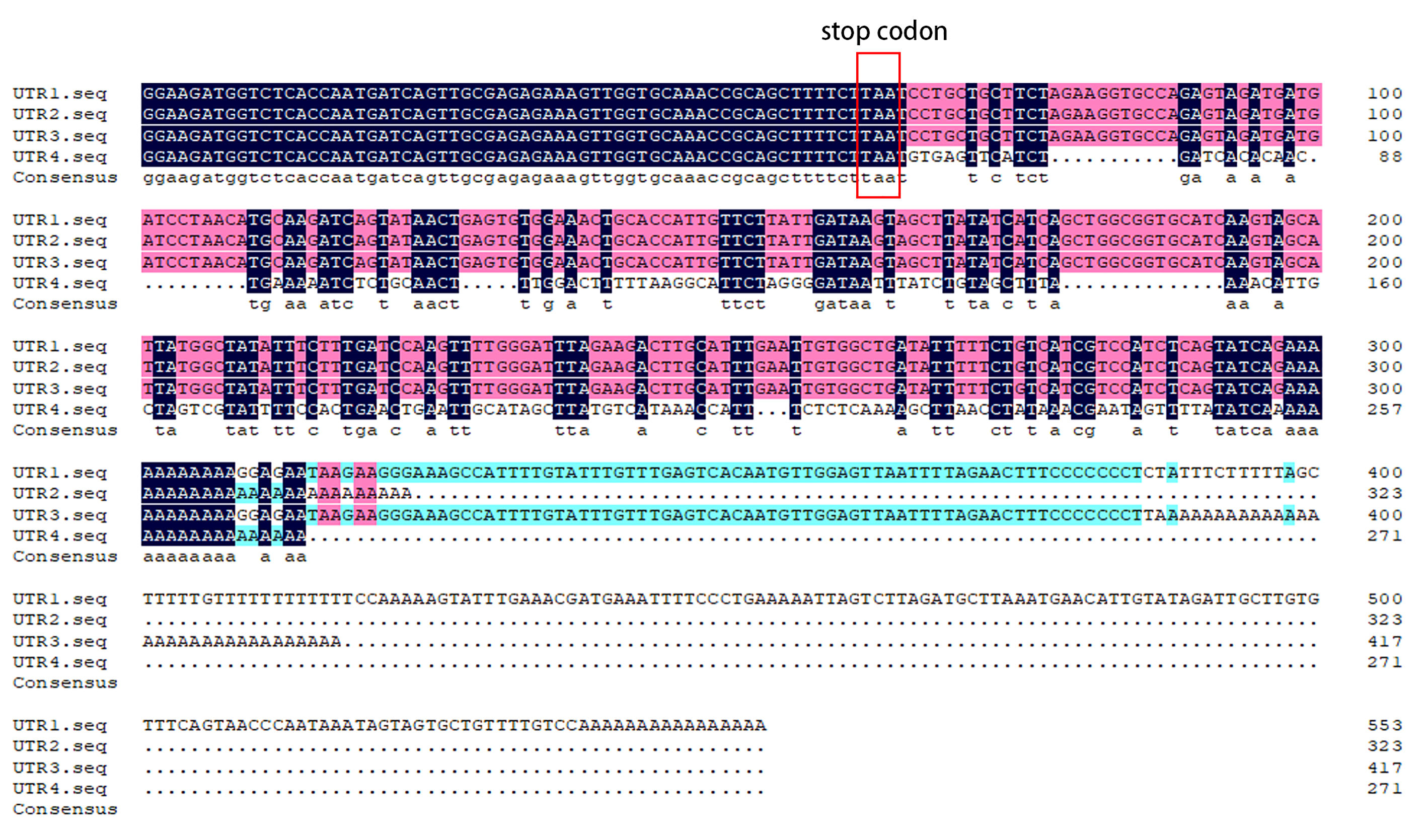

Supplement: Supplementary file 1 — Supplementary Material 1 [file 12864_2023_9696_MOESM1_ESM.jpg]

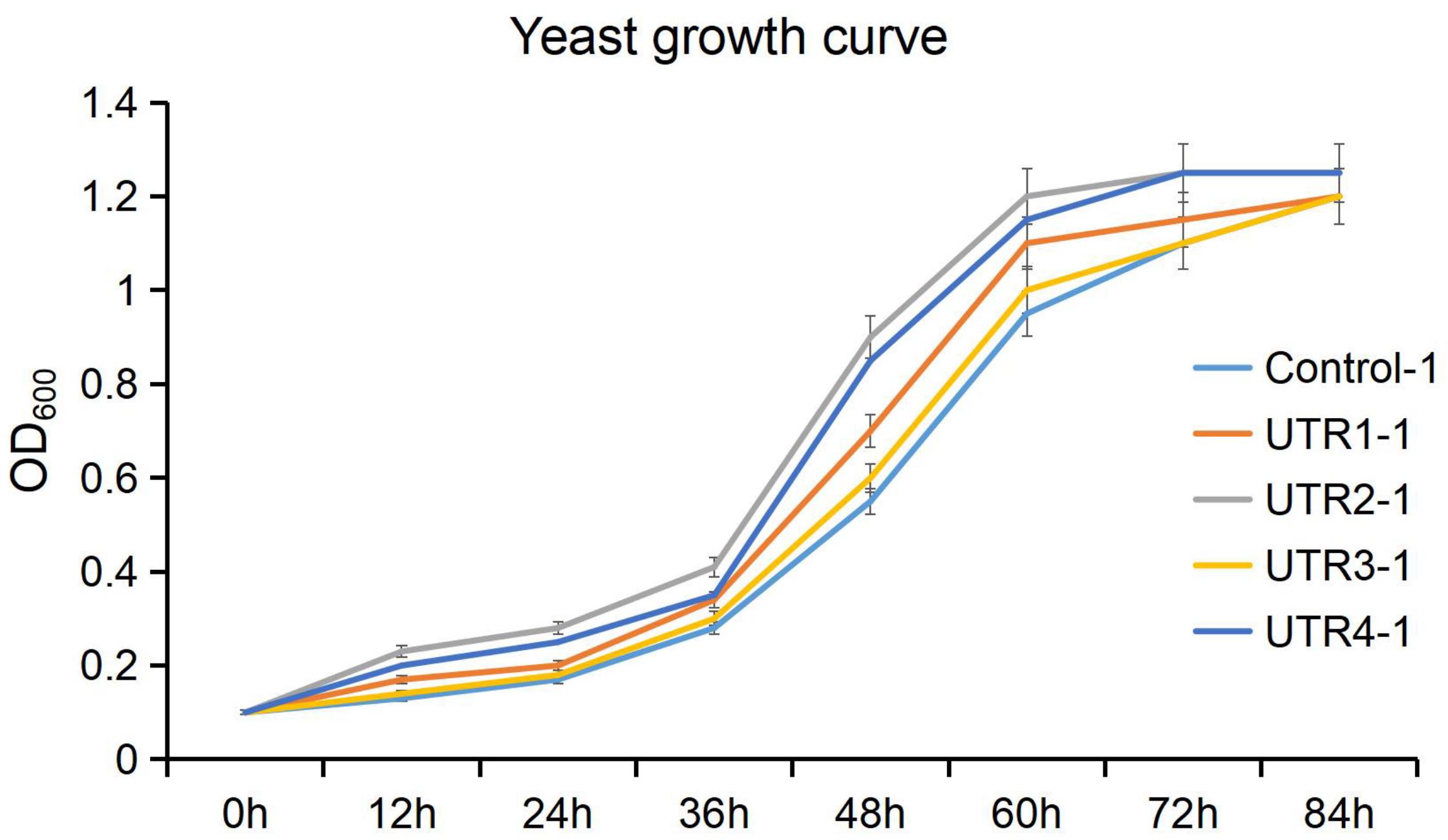

Supplement: Supplementary file 2 — Supplementary Material 2 [file 12864_2023_9696_MOESM2_ESM.jpg]

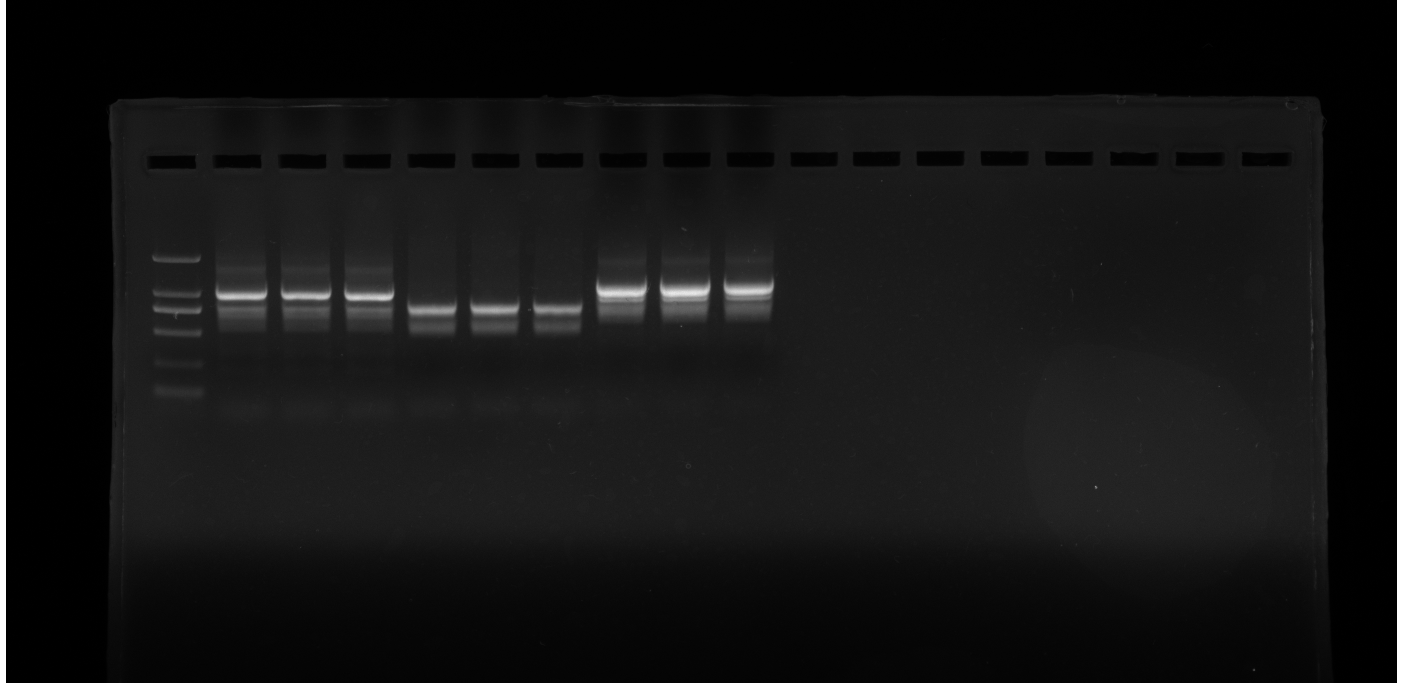

Supplement: Supplementary file 3 — Supplementary Material 3 [file 12864_2023_9696_MOESM3_ESM.png]

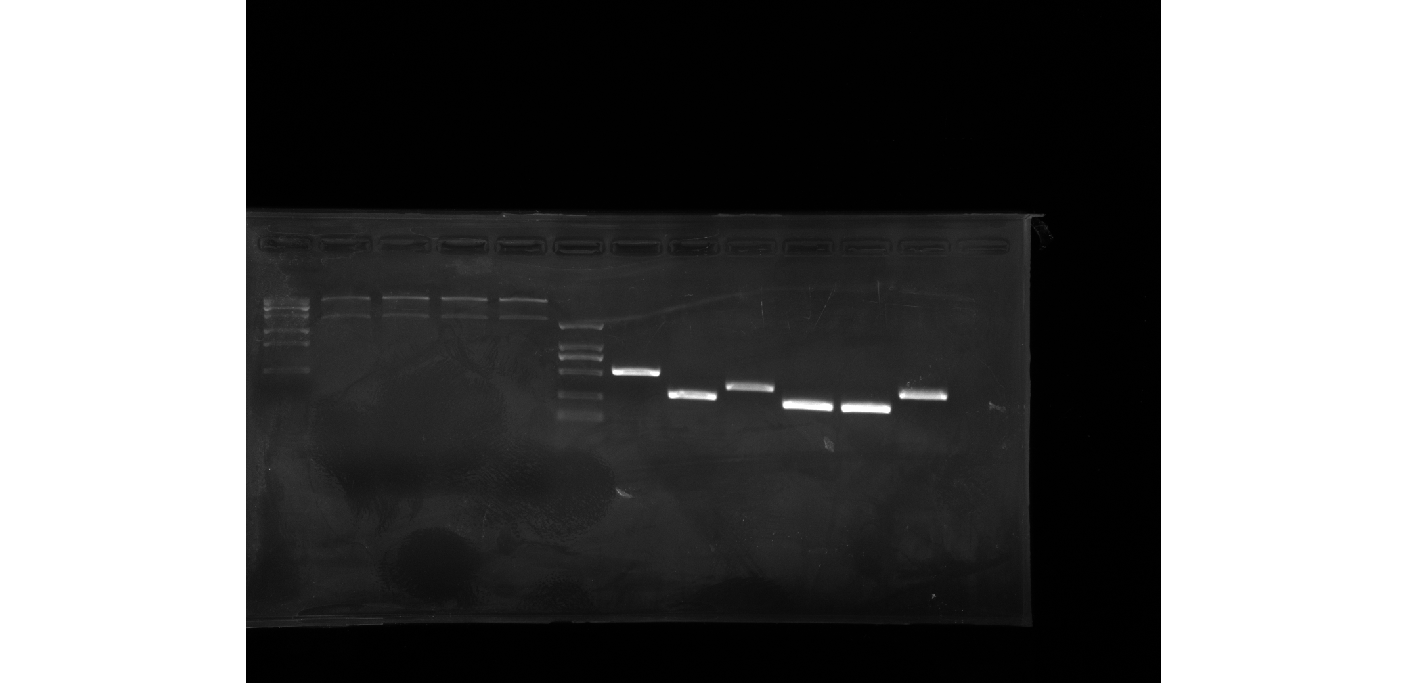

Supplement: Supplementary file 4 — Supplementary Material 4 [file 12864_2023_9696_MOESM4_ESM.png]

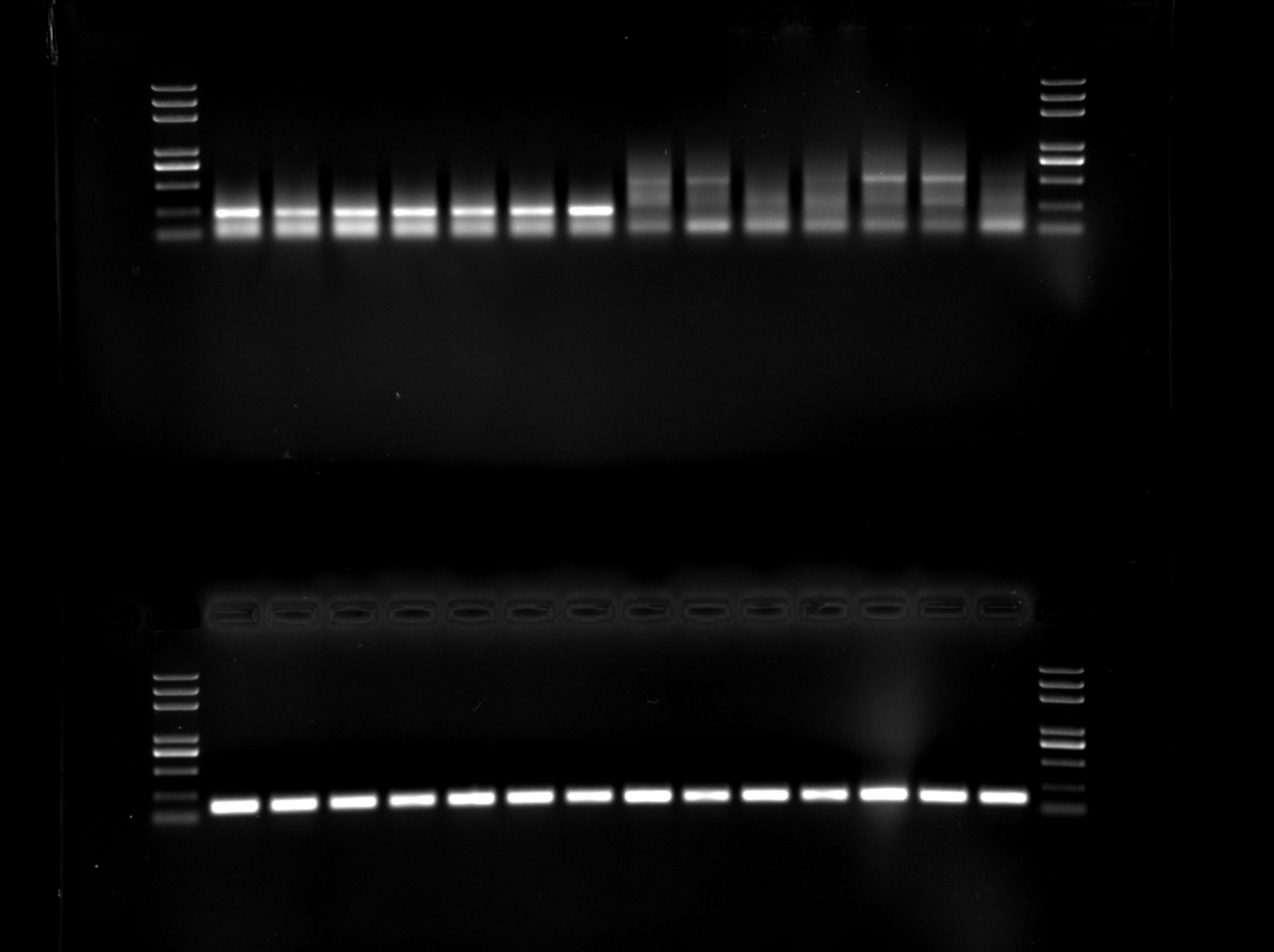

Supplement: Supplementary file 5 — Supplementary Material 5 [file 12864_2023_9696_MOESM5_ESM.png]
